# Do Different Amounts of Exogenous Surfactant Differently Influence Cerebrovascular Instability in a Consecutive Group of Preterm Babies? Preliminary Results from a Single-Center Experience

**DOI:** 10.3390/children11091088

**Published:** 2024-09-05

**Authors:** Andrea Calandrino, Samuele Caruggi, Francesco Vinci, Marcella Battaglini, Paolo Massirio, Gaia Cipresso, Chiara Andreato, Giorgia Brigati, Alessandro Parodi, Giulia Polleri, Diego Minghetti, Luca Antonio Ramenghi

**Affiliations:** 1 Department of Neuroscience, Rehabilitation, Ophthalmology, Genetics, Maternal and Child Health, University of Genoa, 16132 Genoa, Italy; andrea.calandrino@edu.unige.it (A.C.);; 2 Neonatal Intensive Care Unit, Department of Maternal and Neonatal Health, IRCCS Istituto Giannina Gaslini, 16147 Genoa, Italy

**Keywords:** surfactant, prematurity, RDS, NIRS, neuroprotection, cerebral vascular instability

## Abstract

Background: Thirty years ago, the first attempt by Saliba and colleagues was made to reduce the negative effects (hypercarbia) of exogenous surfactant (ES) by slowing its administration. Sixteen years later, we observed the first less invasive surfactant administration (LISA) attempt by Kribs and colleagues. Many studies, since that time, have tried to minimize the invasiveness of ES and subsequent cerebral blood flow perturbations through studies using near-infrared spectroscopy (NIRS). We sought to address this medical challenge by identifying a less problematic modality of ES administration by delivering multiple aliquots of ES instead of a single one, as typically performed. The aim of this study was to test the hypothesis that a different way of administering ES using more aliquots could be a safe alternative that should be assessed in further studies. Methods: Patients between 26 + 0 and 35 + 6 weeks of gestational age (GA) requiring ES administration were enrolled (April 2023–February 2024). Differently fractioned doses were delivered according to an arbitrary standard dosage (0.3 mL per aliquot in babies < 29 weeks; 0.6 mL in babies ≥ 29 weeks), while NIRS and transcutaneous CO_2_ (tCO_2_) monitoring were always performed. ES’s effectiveness was assessed based on the reduction in the Oxygen Saturation Index (OSI) after administration. Persistent desaturation, bradycardia, and airway obstruction were defined as adverse effects and used to evaluate safety during ES administration, as well as variability in NIRS-rSO_2_ values and tCO_2_. Results: Twenty-four patients were enrolled with a median GA of 29 weeks (IQR 4.5) and BW of 1223 ± 560 g. In addition, 50% of the cohort received fewer than three aliquots, whereas the other 50% received more than three. Monitoring was started before the procedure and continued 30′ after the last ES aliquot administration. The variability in NIRS-SpO_2_ values was significantly higher in the group (*p* = 0.007) with a lower number of aliquots administered. Similarly, increased NIRS-rSO_2_ values (*p* = 0.003) and increased tCO_2_ levels (*p* = 0.005) were observed in infants who underwent an ES split after the administration of a low number of aliquots. Conclusions: Our data obtained from the group with > 3 fractionated doses of ES seem to justify the preparation of a more robust study, as the combination of reduced NIRS variability and reduced tCO_2_ maximum levels is consistent with more stable cerebral blood flow during the challenging time of ES administration.

## 1. Introduction

The rise of exogenous surfactant (ES) therapy has revolutionized neonatal care, offering a lifeline to countless infants affected by respiratory distress syndrome (RDS) by improving survival and reducing pneumothorax occurrence [1,2,3].

ES administration by endotracheal tube (ET) is usually carried out in a single bolus in most centers [3,4,5].

We are aware that single bolus administration can lead to obstruction of the ET, with the possible occurrence of acute distention of terminal airways, hypercarbia, air-leak syndromes, severely compromised circulation, and intraventricular hemorrhage [6,7].

For all the abovementioned reasons, due to the lack of explicit consensus on the preferred delivery modality, we acknowledge a significant inter-center difference in ES administration, and ES may be split into smaller aliquots to reduce its potential negative impact [8,9,10,11].

While ES therapy significantly improves respiratory outcomes, some authors recommend managing the procedure to minimize potential hazards to clinical stability, which is crucial in the first hours of life to protect these babies’ immature brains [6,12].

The administration of ES is associated with a reduction in mean arterial blood pressure and an increase in PaCO_2_ levels, as well as cerebral vasodilatation, and a decrease in the left-to-right shunt across the patent ductus arteriosus (PDA), with subsequent system hypovolemia and hypoxia [13].

In the last thirty years, several studies have tried to highlight the complex impact of exogenous surfactant administration on cerebral blood perfusion, employing emerging neuromonitoring tools such as cerebral blood flow velocity (CBFV) through transcranial Doppler ultrasound [14,15], near-infrared spectroscopy (NIRS) [16,17,18,19], and electroencephalogram (EEG) [20,21].

The results of these studies support the hypothesis that ES administration, even if administered with different modalities and velocities, can induce acute changes in cerebral blood flow (CBF), resulting in possible germinal matrix–intraventricular hemorrhage (GMH-IVH) or cerebellar hemorrhage (CBH) incidence [12,13].

On the other hand, the introduction of new, less impactful modalities, like less invasive surfactant administration (LISA), which was first attempted by Kribs et al. in 2010 [22], represents a very promising solution to overcome this challenge.

Despite these efforts, we believe there still a need for an agreement on which method of surfactant instillation would have a lower impact on cerebral circulation [23].

This research aims to assess the safety of a fractioned dose of ES in a monocentric cohort of preterm babies and to compare whether the expected beneficial effect of a smaller volume of fluid administered into the lungs is compromised by increasing the number of procedures.

## 2. Materials and Methods

We conducted a comparative observational study involving all newborns born at IRCCS Istituto Giannina Gaslini, Italy, between 26 + 0 and 35 + 6 weeks of gestational age (GA) and subsequently admitted to the Neonatal Intensive Care Unit (NICU) during the period April 2023–February 2024 with an RDS diagnosis and the need for ES. Infants born at lower GAs were excluded because of the higher risk of developing GMH-IVH during care and maneuvers, as well as the possible need for precocious ES administration during delivery room assistance. Furthermore, infants below 26 weeks of GA at birth experience higher clinical instability and have the propensity to develop skin lesions as a result of the application of the sensors. We considered the first drug administration, which was made by using a PVC ET whose size was chosen according to GA, under continuous multimodal monitoring, which included peripheral pre-ductal saturation (SpO_2_), transcutaneous carbon dioxide tension (tCO_2_), and cerebral regional saturation (rSO_2_). Values were registered by applying a NIRS detector on the newborns’ foreheads. Derived cerebral fractional tissue oxygen extraction (cFTOE), a helpful index to assess cerebral regional oxygen extraction and perfusion, was also obtained for all records using the following formula [24]:

(1) $$\frac{{\mathrm{SpO}}_{2}-{\mathrm{rSO}}_{2}}{{\mathrm{SpO}}_{2}}$$

NIRS monitoring was performed using the MASIMO ROOT system (Masimo Corporation, Irvine, CA, USA); a MASIMO Radical 7 device was used to monitor peripheral blood oxygen saturation (SpO_2_), whereas a MASIMO O_3_ sensor was used to monitor cerebral regional oxygen saturation (rSO_2_). The MASIMO O_3_ sensor provides readings every 2 s [25]. It has been approved for use in pediatric patients. It has a reported bias, standard deviation (SD), standard error (SE), and root-mean-square error (RMSE) for rSO_2_ of 2.6%, 4.5%, 0.3%, and 4.3%, respectively, and a trend accuracy with a relative mean error of −1.4%, an SD of 4.3%, an SE of 0.2%, and an RMSE of 3.9% [24].

Levels of tCO_2_ were registered through a Sentec transcutaneous monitoring system (Sentec AG, Therwil, Switzerland) by registering transcutaneous PaCO_2_ levels every 60 s.

We collected data only from patients who underwent monitoring from 15 min before the beginning of endotracheal surfactant administration to 30 min after the end of the procedure.

We excluded patients who received surfactant administration during delivery room resuscitation and repeated ES administrations due to the smaller amount of surfactant (usually 100 mg/kg).

The other exclusion criteria included individuals with congenital pulmonary pathologies, such as congenital diaphragmatic hernia and pulmonary malformations, and those with congenital heart diseases.

The diagnosis of RDS was based on clinical and radiological criteria [3,26], namely, a Silverman Score > 4, an oxygen requirement (FiO_2_) ≥ 0.3, and a diffuse reticule-granular pattern on chest radiography [27].

As per the internal protocol of our center, surfactant was administered through ET instillation using a closed sterile circuit [28] at a dose of 200 mg/kg. Differently fractioned doses were delivered, with a free time interval between each dose of at least 3 min, according to an arbitrary standard dosage (0.3 mL per aliquot in babies ≤ 29 weeks; 0.6 mL in babies > 29 weeks), as determined by the attending Neonatologist.

To assess the effectiveness of surfactant administration, a decrease in Oxygen Saturation Index (OSI) [29] 12 h after therapy of at least 10% was considered [30].

We defined as an adverse effect the registration of persistent desaturation during or after the administration of ES (SpO_2_ values below 80% for more than 5 min after ET administration) and the presence of bradycardia (heart rate below 90 bpm for more than 60 s after ET administration) [6]. Also, persistent airway obstruction (not resolved by ventilation) and pulmonary hemorrhage were considered [7].

For each infant, we collected data about sex, gestational age, birth weight, perinatal history, blood gas analysis at birth, the development of low-grade intraventricular (GMH-IVH) or cerebellar (CBH) hemorrhages, and the number of surfactant aliquots administered.

Serial cranial ultrasound examinations were performed according to the routine protocol in our NICU, which includes studies at days 1, 2, 3, and 7 after birth and subsequently weekly until term-corrected age. All cranial ultrasound examinations were performed through the anterior and the mastoid fontanel using an 8 Mhz convex probe (Venue™ point-of-care ultrasound system, GE HeatlhCare Ltd., Little Chalfont, UK). MRI studies, including SWI sequencing [31,32], were performed on a 3T scanner (Siemens, Enlargen, Germany) using a dedicated neonatal head array coil [33]. The susceptibility-Weighted Imaging (SWI) parameters were as follows: repetition time/echo time = 33 ms/48 ms; flip angle = 15°; field of view = 120 mm × 120 mm; matrix size = 152 × 137; total acquisition time = 2 min 57 s.

Descriptive statistics were generated for the whole cohort; the data were expressed as mean and standard deviation (SD) or median and interquartile range (IQR) for continuous variables and as absolute and relative frequencies for categorical variables. Variance values (σ^2^) were calculated and expressed for data derived by multimodal monitoring and z-scores, which were estimated to express the width of the data ranges.

The normality of distribution for all variables was assessed graphically or using the Kolmogorov–Smirnov R test, where appropriate. Comparisons of categorical variables among subgroups were carried out using Fisher’s exact tests, while for continuous variables, a Mann–Whitney U test or a Student’s *t*-test was selected.

Comparisons of rough and derived data (SpO_2_, tCO_2_, rSO_2_, CFTOE) obtained from multimodal monitoring, then divided into subgroups, were performed with one-way ANOVA tests. A *p*-value of < 0.05 was considered statistically significant, and all *p*-values were based on two-tailed tests. Statistical analysis was performed using the Jamovi project interface software (v. 2.3.28) based on the R language for statistical computing.

## 3. Results

Of all 153 babies born prematurely (GA < 37 weeks) in the considered period, ES was necessary in 42 patients according to our clinical criteria. Of these patients, we excluded ten patients who were born at a GA < 26 weeks and three patients who were administered ES during delivery room resuscitation. A lack of complete monitoring data was reported in five cases, which were excluded. No cases of congenital cardiac or pulmonary malformations were reported.

A total of 24 patients were included. The median GA was 29 weeks (IQR 4.5), and the mean birth weight (BW) was 1223 ± 560 g. The population’s clinical characteristics are reported in Table 1, where patients were divided according to a gestational age cut-off of 29.

No significant differences were found in terms of perinatal history and post-natal adaptation.

Only two cases required ES to be administered through ET following the InSurE technique [10], while prolonged mechanical ventilation was necessary in all the other cases.

For data analysis, we divided the whole cohort into two subgroups equivalent in number, according to a predetermined quantity of less or more than three aliquots of ES administered. ES delivery was defined as effective in 22 patients (92%), no detectable effects were registered in 2 cases (8%), and no adverse events were registered in any. In particular, the mean OSI starting value (before ES) of the < 3 aliquots subgroup was 5.3, and it was 3.9 in the ≥ three aliquots one (*p* = 0.2). The mean index improvement 12 h after ES administration showed a mean reduction of 2.6 in the first group and 2.4 in the latter (*p* = 0.8).

Brain MRI, through the SWI sequence, detected two low-grade GMH-IVHs (one also detected by cranial US) and two punctate CBHs. In one case, we reported bilateral periventricular leukomalacia (PVL) in a patient at 33 + 0 weeks of GA, detected either during brain US screening or after brain MRI (Table 1).

The results of the multimodal monitoring are presented in Table 2. Specifically, a significant difference was not detectable in the average values of each monitoring. At the same time, this was evident in the σ^2^ values, suggesting that the fluctuations in each parameter were not comparable between the two sub-cohorts, with significant wider registered variation in the group administered a less fractioned dose of ES. Considering the tCO_2_ registrations, we report a difference in the mean values during the global registration period, with significantly higher values in the group that received less fractioned ES administration; the σ^2^ values also confirm this trend. We did not confirm this difference in the baseline values (before the procedure). At the same time, we registered a significant difference in the maximum values reached (61.8 mmHg in the less fractioned group vs. 47.2 mmHg in the more fractioned one, *p* = 0.005), with a return to the baseline values in both the sub-cohorts at the end of the monitoring period, suggesting that slight hypercarbia occurred in the group administered a less fractioned dose.

To confirm the above hypothesis, we calculated the range of values obtained for each monitoring and expressed them as z-scores; in Table 3, we show that a significant difference in the range width (named Δ) is reported for all variables except for cFTOE.

## 4. Discussion

We have shown that fractioned ES administrations are safe, as no adverse events were registered during the study period. More interestingly, patients who received < 3 aliquots of ES, which were 0.3 mL larger in volume if compared to the other group, were associated with increased variation in SpO_2_, rSO_2_, cFTOE, and tCO_2_ values compared to babies receiving smaller but more numerous ES administrations, suggesting that higher cerebral vascular instability may take place with higher volumes of ES. These data were inconsistent with the ΔcFTOE calculation; our conclusion remains uncertain probably due to the relatively small sample size for a mathematically derived variable ranging between 0 and 1.

Variation in rSO_2_ in extremely preterm infants may indirectly reflect more vulnerable cerebral autoregulation, as well as increased cFTOE oscillation, which reflects the balance between cerebral oxygen supply and cerebral oxygen consumption, expressing variability either in oxygen supply or oxygen delivery efficiency [24].

To our knowledge, this is the first study assessing cerebral NIRS variation monitoring. In a study from Saliba [20], the authors demonstrated that a slower instillation of ES induced no changes in CBFV studied with Doppler, unlike rapid instillation in a cohort of extremely preterm newborns, paving the way for new modes of administration characterized by lower level of invasiveness.

In particular, we reported that a more fractioned dosage, which, in theory, may expose infants to more numerous stressful events during administrations, has a minor impact on cardiorespiratory and cerebral homeostasis, supporting the idea that this approach could be included in a bundle of care aiming to better protect the immature brain [34,35].

In recent years, research has focused on comparing the two most widespread techniques, LISA and InSurE, showing contrasting data regarding efficacy and cardiovascular impact [36,37,38].

On the other hand, few studies have been produced on surfactant fractioning, although several NICUs use this method [13,14,16,17,22,23]. To date, surfactant fractioning has been studied mainly on animals, with inconsistent results [21,39]. A 1993 study by Zola et al. [11] demonstrated the equivalence in terms of efficacy between administering an entire bolus or fractions, allowing us to design our study.

Despite the increase in transcutaneous carbon dioxide in babies receiving higher volumes of ES not reaching statistical significance, this trend may suggest that alveolar exchange capability may be affected by the presence of more significant amounts of fluid in a unit of time.

Although again somewhat dated, several studies have attempted to understand PaCO_2_ variations related to different modalities of ES administration, either in animals [39] or humans [21]. Particularly, Lundstorm et al. [21] attempted to demonstrate the effect of CO_2_ variations by splitting the prescribed surfactant dose into different numbers of aliquots (namely 2 and 6), revealing a transient slower variation in blood gas concentration. These results contrast with ours, where we tried to preliminary demonstrate the protective role of fractioned administrations and a lack of adverse events. A possible explanation for this discordance may be found in the interval time among the administrations, which the authors did not specify, and which may cause a volume overload if it is not long enough to allow drug absorption from the alveolar interface.

Another possible explanation for our findings is that, according to Hentschel et al. [6], the administration of more significant volumes of ES may lead to obstruction of the ET, the tracheobronchial tree, or drug reflux in the upper airways, causing a quick slight rise in PaCO_2_ levels [6,7,20]. Furthermore, clinicians are forced to increase the peak inspiratory pressure to resolve obstructions, leading to enlargement of the terminal airways, pneumothorax, and air leak syndrome, further compromising cardiovascular homeostasis [6]. Accordingly, air leak syndrome [40] and hypercarbia [41] are well-known risk factors for severe brain damage in premature infants due to the acute perturbation of cerebral blood flow, which could lead to the development of GMH-IVH or CBH [24,41,42].

No statistical significance was found in the two groups for GMH-IVH and CBH incidence. The child who developed PVL was a patient with severe fetal anemia and perinatal asphyxia requiring resuscitation, with an APGAR score of 5–7 and acidosis at birth (pH 6.9, BE−17).

The cornerstone of a compelling bundle of care aims to avoid potential stressor events promoting cerebral vascular instability and known insults that lead to brain damage development. The application of NIRS monitoring in the first days of life, especially during invasive procedures, has shown perturbation in cerebral blood flow, potentially harmful to preterm newborns [24,37,38,41,43,44,45].

Notably, several studies have highlighted the presence of correlations between alterations in rSO_2_ and cFTOE and IVH development in different cohorts of premature babies [24,43,44,45], with some interesting implications, as proposed by Ashoori et al. [46], who developed an effective machine learning model on NIRS monitor data able to predict the occurrence of IVH.

In addition, the higher baseline and mean tCO_2_ associated with lower aliquots of ES may put infants at risk of a condition once called “permissive hypercapnia,” a strategy allowing for mildly elevated levels of carbon dioxide (45–55 mmHg) to minimize ventilation-induced lung damage [47]. This strategy is known to be detrimental to infants less than 750 g in birth weight [48] and is not exactly a reassuring neuroprotective strategy [49].

All the discussed measures align with the principles of minimal handling, insults, and variations in hemodynamic stability, which are fundamental to preventing GMH-IVH occurrence. Therefore, reducing all potential stressors related to medical and nursing care is of utmost importance in managing premature infants, especially during the first hours and days of life [35,38,39,40,50,51].

The limitations of the present study are the small size of our cohort, the exclusion of the lowest-gestational-age babies, the higher risk of brain damage, and no other ES administration technique being considered for comparison. Moreover, per our internal protocol, the two obtained sub-cohorts contained patients with different characteristics from a GA and BW point of view and who received different volumes of ES. We acknowledge that this may represent a confounding factor since we already know from several studies in the literature that both rSO_2_ and cFTOE are influenced by GA, arterial pressure, gender, and being born small for gestational age (SGA) [52,53,54]. Additionally, regarding these parameters’ variability, Alderliesten et al., 2016 [54] underline how average rSO_2_ increases with GA (1% per week) and follows a parabolic curve in relation to postnatal age, with a peak at ~36 h, without showing more significant variability when following this trend. In another study by Massirio et al., 2024 [24], NIRS monitoring was implemented in preterm patients undergoing extubation; the patients were divided into similar groups to ours, without showing any variability. On the other hand, as already highlighted, impaired cerebrovascular autoregulation is associated with lower gestational age [54], but it has been shown [55] that fluctuations in CO_2_ are associated with fluctuations in cFTOE and rsO_2_ values, and we confirmed this association in this paper. In addition, the mean gestational age of 29 weeks makes this gestational age group not at such a high risk of developing GMH-IVH, with the most important outcome to be protected below 28 weeks [32,33]. More studies involving a larger cohort of patients, especially preterm patients, are necessary to confirm the hypotheses raised above, and an MRI follow-up is needed to assess the actual incidence of even minor bleeds following the more prudent administration of ES.

## 5. Conclusions

These preliminary data aim to assess the effects of fractionated surfactant administration through ET in a cohort of premature infants, considering NIRS monitoring variations and tCO_2_ trend monitoring. The lack of adverse events encourages the design of future randomized clinical trials to compare the impact of different ES administration modalities on cerebral vascular stability.

Cerebral hemodynamic and chemical equilibrium express more significant fluctuations related to less drug splitting, highlighting the necessity of more fractioned ES administration to limit blood gas variations and the subsequent impact on cerebral hemodynamics, together with many other precautions we practice in our unit, precautions aiming to minimize insults related to the development of GMH-IVH [34,35].

Any effort to contain these effects and achieve more stable cerebral oxygenation during surfactant administration could be one of the keys to reducing the potential harm to the central nervous system, consistent with those bundles of care aiming to facilitate the development of GMH-IVH.

## Figures and Tables

**Table 1:** Patients’ main clinical and demographic characteristics.

	<29 Weeks	≥29 Weeks	*p*-Value
**# of patients (%)**	11 (46)	13 (54)	0.68
**Gestational age (median; IQR)**	26; 3	31; 4	**<0.001**
**Birth weight (mean ± SD)**	784 ± 250 g	1594 ± 473 g	**<0.001**
**APGAR 1′ (median; IQR)**	5; 4	6; 3	0.14
**APGAR 5′ (median; IQR)**	8; 2.5	8; 0	0.25
**UA pH at birth (mean ± SD)**	7.21 ± 0.07	7.19 ± 0.12	0.17
**UA BE at birth (mean ± SD)**	−6.2 ± 1.5	−8.6 ± 4.3	0.08
**Low-grade GMH-IVH (US/MRI)**	1/2	0	0.25 *
**CBH**	0/1 (punctate lesions)	0/1 (punctate lesions)	1 *
**Adverse events during/after ES**	0	0	1

#, number; SD, standard deviation; IQR, interquartile range; UA, umbilical artery; BE, base excess; GMH-IVH, germinal matrix/intraventricular hemorrhage; CBH, cerebellar hemorrhage; US, detected by cranial ultrasound; MRI, detected by brain MRI; ES, exogenous surfactant. * *p*-value calculated considering the gold standard methodology of brain MRI.

**Table 2:** Multimodal monitoring results of the aliquot-divided sub-cohorts.

	≥3 ES Aliquots (Patients ≤ 29 Weeks of GA)	<3 ES Aliquots (Patients > 29 Weeks of GA)	*p*-Value
**SpO_2_**	92.8%	92.3%	0.8
**σ^2^-SpO_2_**	4.84%	13.69%	**0.006**
**rSO_2_**	76.7%	74.7%	0.6
**σ^2^-rSO_2_**	4.41%	38.44%	**0.002**
**cFTOE**	0.1%	0.2%	0.6
**σ^2^-cFTOE**	4 × 10^−4^%	3.6 × 10^−3^%	**0.05**
**tCO_2_-baseline**	47.2 mmHg	44.1 mmHg	0.4
**tCO_2_-max**	47.2 mmHg	61.8 mmHg	**0.005**
**tCO_2_-after 30′**	42.9 mmHg	50.2 mmHg	0.07
**tCO_2_-mean**	45.00 mmHg	53.4 mmHg	0.06
**σ^2^-tCO_2_**	1.64 mmHg^2^	4.41 mmHg^2^	**<0.001**

SpO_2_, peripheral oxygen saturation; rSO_2_, cerebral regional mixed venous oxygen saturation; cFTOE, cerebral fractional tissue oxygen extraction; tCO_2_, transcutaneous CO_2_; σ, variance.

**Table 3:** The difference in values of all the parameter range measurements.

	≥3		<3		
	Mean Value	Z-Score	Mean Value	Z-Score	*p*-Value
**ΔSpO_2_**	23.3%	0.2	13.2	−0.8	0.007
**ΔrSO_2_**	29.0%	0.4	13.3	−0.7	0.003
**ΔcFTOE**	0.3%	0.3	0.2	−0.7	0.07
**ΔtCO_2_**	11.6 mmHg	0.7	4.3	−0.7	0.002

SpO_2_, peripheral oxygen saturation; rSO_2_, cerebral regional mixed venous oxygen saturation; cFTOE, cerebral fractional tissue oxygen extraction; tCO_2_, transcutaneous CO_2_.

## Data Availability

The data presented in this study are available on request from the corresponding author due to privacy and ethical restrictions.
